# High Prevalence of Occult Hepatitis B Virus Co-Infection Identified in Treponema Pallidum-Positive Blood Donations: Implications for HBV Risk Reduction

**DOI:** 10.3390/pathogens15070776

**Published:** 2026-07-22

**Authors:** Xianlin Ye, Xiaoxuan Xu, Jinfeng Zeng, He Xie, Jujun Sun, Baoren He, Limin Chen

**Affiliations:** 1 Department of Laboratory, Shenzhen Blood Center, Shenzhen 518035, China; yexianlin90@hotmail.com (X.Y.); szxuxiaoxuan@163.com (X.X.); zzengjf@163.com (J.Z.); 2 The Hospital of Xidian Group, Xi’an 710077, China; ixiehe000@163.com (H.X.); sbksjj@163.com (J.S.); 3 The Joint-Laboratory of Transfusion-Transmitted Diseases (TTDs) Between IBT, CAMS and Nanning Blood Center, Nanning Blood Center, Nanning 530003, China; 4 Provincial Key Laboratory for Transfusion-Transmitted Infectious Diseases, Institute of Blood Transfusion (IBT), Chinese Academy of Medical Sciences (CAMS) & Peking Union Medical College (PUMC), Chengdu 610052, China

**Keywords:** Treponema Pallidum (TP), occult hepatitis B virus infection (OBI), blood screening, blood safety, co-infection

## Abstract

Over the past decade, the incidence of infectious syphilis has been on the rise in the general Chinese population. Consequently, Treponema Pallidum (TP) testing has been proposed as a surrogate marker for sexually transmitted pathogens and for monitoring risky sexual behaviors among blood donors globally. In addition, sexual contact with individuals chronically infected with hepatitis B virus (HBV) is recognized as one of the primary routes of HBV transmission. Blood donors may acquire HBV infection through sexual contact with chronically infected partners, particularly with occult hepatitis B infections (OBIs), which are characterized by intermittent and extremely low viral loads. Therefore, the prevalence of OBIs among syphilis-positive blood donations and the corresponding risks to blood safety require further investigation. This study aimed to investigate the prevalence of OBIs among syphilis-positive blood donors and assess the surrogate value of TP testing for evaluating OBI-related risks to blood supply. After routine screening using serological assays and nucleic acid testing (NAT), blood donation samples with positive anti-TP enzyme-linked immunosorbent assay (ELISA) results were collected and further confirmed by the Treponema Pallidum Particle Agglutination Assay (TPPA). For blood donations confirmed positive for syphilis, further tests were performed to characterize whether the donations had HBV co-infection, including electrochemiluminescence immunoassay (ECLI) for the detection of hepatitis B surface antigen (HBsAg), anti-hepatitis B surface antibody (anti-HBs), hepatitis B e antigen (HBeAg), anti-hepatitis B e antibody (anti-HBe), and anti-hepatitis B core antibody (anti-HBc). Additionally, quantitative real-time polymerase chain reaction (qPCR) was used for HBV DNA quantification, and nested PCRs for the S and basal core promoter/precore (BCP/PC) region were conducted in combination with high-volume nucleic acid extraction. Subsequently, molecular characterization of HBV DNA in these co-infected samples was carried out by DNA sequencing to analyze the viral genetic features. Of 252 anti-TP ELISA+ donations screened from 64,871 blood samples, 138 (138/250, 55.2%) donations were confirmed syphilis-positive but NAT−, among which 78 (78/138, 56.5%) were anti-HBc-positive, and 88 (88/138, 63.7%) had anti-HBs. Notably, seven donations (7/138, 5.1%) were diagnosed as OBI co-infections, and available sequence analysis revealed that three cases were genotype B and one case was genotype C. In addition, several mutations in the S region of the HBV genome were identified, including Q101R, K122R, Q129H, T131N, M133T, G145R, and Y161F mutations. Furthermore, nucleotide mutations such as T1719G, A1752T, G1896A, and A1762T/G1764A in the BCP/PC regions were also detected in these OBI donations. These mutations may contribute to the extremely low HBV viral loads and/or failure in HBsAg detection, collectively leading to OBIs. These data indicate that syphilis screening of blood donors has potential to serve as an additional safeguard measure for excluding donations co-infected with OBIs. The high prevalence of undetected OBIs in syphilis-positive blood donors further supports that syphilis screening has the potential to serve as a surrogate marker for HBV-related risks in the blood supply.

## 1. Introduction

Syphilis is an infectious disease caused by TP infection, and unprotected sexual contact and trans-placental spread during pregnancy are its main spreading routes [1]. It is one of the mandatory serological screening tests for transfusion-transmitted infections (TTIs) recommended by the World Health Organization (WHO) [2]. Syphilis is estimated to affect 36 million people worldwide, with 11 million new cases each year and more than 90% occurring in developing nations [3,4]. It was reported that syphilis accounts for approximately 10% of the sexually transmitted diseases [5]. According to the Chinese Health Statistical Digest by the Chinese Ministry of Health, the incidence of syphilis is ranked third following viral hepatitis and tuberculosis in Chinese class A and B communicable diseases [6].

In recent years, syphilis infection has been on the rise in China. The total incidence of syphilis increased from 1.0 to 32.2 per 100,000 between 1995 and 2016 in China [7]. Among blood donors, it has been reported that the seroprevalence of TP antibody ranged from 0.31 to 0.70% in different regions of China [8,9]. This relatively high prevalence of TP infection may endanger blood safety as some of these infected individuals may go to donate blood without realizing that they were actually infected. Therefore, it is necessary to identify the high-risk groups in the blood donor population to further ensure blood safety.

Our previous study observed that the prevalence of occult hepatitis B infections (OBIs) in the partners of chronically infected HBV individuals was significantly higher than that in the general donor population, and the majority of these OBIs could not be detected by routine screening with ID NAT and HBsAg, indicating a potential risk to blood safety [10]. As both are sexually transmitted pathogens, blood donors may be co-infected with HBV and TP. To clarify the epidemiological profile of TP and hepatitis B virus co-infection in blood donors and to assess the associated risk to blood safety, we enrolled a large cohort of Chinese blood donors in Shenzhen Blood Center. All participants were screened for HBV and TP using serological and molecular assays. For TP-seropositive donors, high-volume nucleic acid extraction was applied to detect HBV DNA as previously reported [10], so as to evaluate the potential risk of screening failure to blood safety.

## 2. Materials and Methods

### 2.1. Blood Samples and Routine Screening

A total of 64,871 blood donations were collected by the Shenzhen Blood Center between August 2023 and January 2024. Demographic data, including gender, age, educational background, and occupation, were routinely collected from donors. Before donation, informed consent, the donation registration form was signed, and all donors passed the physical examination and health questionnaire, as well as rapid tests for HBsAg, ALT, and hemoglobin (Minray Bio-Medical Electronics, Ltd., Shenzhen, China) [11]. Routine screening with dual ELISA assays for HBsAg and anti-HIV (Bio-Rad Laboratories, Inc., Hercules, CA, USA and Zhuhai LivZon Diagnostics Inc., Zhuhai, China); anti-HCV and TP (WanTai Diagnostics Inc., Beijing, China and Zhuhai LivZon Diagnostics Inc., Zhuhai, China) was performed. If both results for TP screening were reactive, the sample was classified as TP-negative. Conversely, if one test yielded a negative result while the other was reactive, the sample was retested in duplicate using the same assay kits. If one or two of the repeated results were positive (repeat reactive), the sample was defined as TP ELISA-positive. All TP ELISA-positive samples were collected and stored for further testing. In addition, all donations were screened by NAT for HBV DNA, HCV RNA, and HIV RNA using the Procleix^®^ Ultrio Elite^®^ Assay (Novartis Diagnostics, Emeryville, CA, USA) on the Elite platform and discriminatory strategies were implemented as reported in our previous study [12]. The routine screening data of blood donors, covering the entire workflow from donor recruitment to the issuance of test results, were archived in the electronic system to facilitate retrospective analysis. Further study-specific laboratory testing, data extraction, and statistical analysis were initiated after 7 February 2024.

### 2.2. TP Confirmation

To confirm TP infection in ELISA anti-TP-positive donations, confirmatory tests by TPPA and Toluidine Red Unheated Serum Test (TRUST) were performed by the Shenzhen Center for Chronic Disease Control and Prevention. Donations with TPPA+ and TRUST + were confirmed as TP-positive.

### 2.3. Supplemental Serologic Testing and NAT Testing

TP-positive donations were further analyzed by HBsAg II Quant, along with anti-HBs, HBeAg, anti-HBe, and anti-HBc by ECLI (Roche Elecsys, Indianapolis, IN, USA) [12]. Additionally, TP-positive samples were further tested individually by MPX2.0 ID HBV NAT (Roche Diagnostics, Pleasanton, CA, USA; LOD: 2.3 IU/mL; input volume 1 mL). Molecular assays for donations with confirmed TP-positive results were performed using a combination of qPCR and nested PCRs that amplify the BCP/PC and the S regions with a large extraction volume of 2.5–5 mL plasma in order to detect very low-level OBIs. HBV DNA sequencing, serotyping, and genotyping were analyzed as previously reported [13,14]. The sensitivity and specificity of the tests used in this study were summarized in Table 1.

### 2.4. OBI-Associated Residual Risk (RR) in Blood Donations Without Anti-TP Screening

According to a previous study [15], the RR of OBI without anti-TP screening was calculated as follows:

RR = *p* (OBI without anti-TP screening) × *p* (transmission rate)

### 2.5. Statistical Analysis

The statistical software STATA 16.0 was used for data analysis. Categorical variables and continuous variables were analyzed using Fisher’s exact test and the non-parametric Mann-Whitney test, respectively. The 95% confidence intervals (CIs) for the residual-risk estimate were calculated using the Wilson score method. A *p*-value < 0.05 was defined as statistically significant.

## 3. Results

### 3.1. Screening and Detection of Syphilis

**Figure 1:**
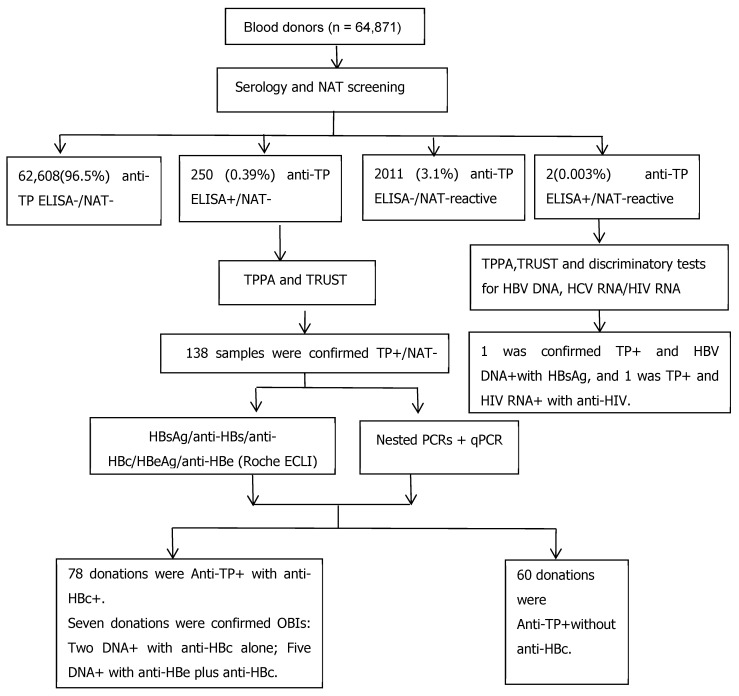
Flow chart of the study design. Dual ELISA assays and NAT were employed as initial screening, while TPPA and TRUST were performed for TP confirmatory tests. Additional HBV serological testing (ECLI) and molecular testing (qPCR and nested PCRs) were used to further clarify OBIs.

A total of 64,871 blood donations were screened by the dual ELISA assays for HBsAg, anti-HCV, anti-HIV, and anti-TP, in combination with NAT for HBV DNA, HCV RNA, and HIV RNA; 62,543 donations were qualified and released, and 252 anti-TP ELISA+ samples were identified, among which 250 were NAT−, one was HBV NAT-reactive with HBsAg ELISA+, and one was HIV NAT-reactive with anti-HIV ELISA+. After anti-TP confirmatory tests and the discriminatory assay for NAT-reactive donations, 138/250 (55.2%) anti-TP ELISA+/NAT− samples were confirmed TP-positive, and one HBV/TP co-infection and one HIV/TP co-infection were identified. Totally, 140 donations were confirmed as TP-positive. Of 138 confirmed TP-positive donations with NAT-negative results, anti-HBc was detected in 78 cases (78/138, 56.5%), while anti-HBs positivity was observed in 88 cases (88/138, 63.7%), as detailed in Figure 1. Notably, seven (7/138, 5.1%) were diagnosed as occult HBV co-infections by various serological and molecular assays.

The demographic, serologic, and molecular characteristics of 138 TP-confirmed positive donations with NAT-negative results stratified by sex are shown in Table 2. For these 138 TP-confirmed positive donations, seropositive rate of anti-TP in first-time donors was higher than that in repeated donors. The prevalence of syphilis was lower (0.19%) in male donors than in female donors (0.25%). We also found that the majority of the TP-confirmed positive donations were in the 45–60 age group, accounting for 43.48%. Our data also indicated that donors with a lower education level (below high school) had a higher proportion of TP infection.

### 3.2. OBI and Anti-HBc Detection in Different Age Groups of TP-Confirmed Positive Donations

A total of 138 confirmed TP-positive donations were screened from 64,871 donations. Specifically, 82 were detected in male donors (82/42,101, 0.19%), and 56 cases were identified in female donors (56/22,770, 0.25%) (Table 2). There were significant differences in gender distribution across different age groups (χ^2^ = 31.1, *p* < 0.0001). Seventy-eight donations were anti-HBc-positive (56.5%; 95% CI: 47.8 to 64.9%) (Table 3). There was a clear increase in anti-HBc prevalence with age, ranging from 9.53% in donors aged under 30 years to 85.19% in those over 50 years (χ^2^ = 31.2, *p* < 0.0001). Six OBIs were detected in the 41–50 age group, and OBI prevalence was significantly higher in this age group than in other age groups (χ^2^ = 8.49, *p* < 0.05).

### 3.3. OBI-Associated RR in Blood Donations Without Anti-TP Screening

In this study, *p* (OBI without anti-TP screening) was seven cases in 62,543 donations, and *p* (transmission rate) can be estimated as 2/11 [16], so the RR was calculated as follows:

RR = *p* (OBI without anti-TP screening) × *p* (transmission rate)

RR = 7/62,543 × 2/11 = 1:49,141 [95% CI: 1:29,291–1:88,495]

### 3.4. Serologic and Molecular Characteristics of Seven Donations Co-Infected with OBI and TP

The HBV s gene was successfully amplified from four co-infected donations, and sequence analysis revealed that three cases were genotype B and one case was genotype C. The viral loads of these donations were less than 10 IU/mL or undetectable. In addition, several mutations in the S region of HBV were identified, including Q101R, K122R, Q129H, T131N, M133T, G145R and Y161F mutations. Furthermore, the BCP/PC region of the HBV genome was successfully amplified from five co-infected donations, and nucleotide mutations such as T1719G, A1752T, G1896A, and A1762T/G1764A in the BCP/PC regions were also detected (Table 4).

## 4. Discussion

Blood transfusion is critical for saving the lives of patients. Nevertheless, it also bears the risk for transmission of blood-borne pathogens such as HBV, hepatitis C virus (HCV), human immunodeficiency virus (HIV) and TP. In this study, we particularly focused on the prevalence of TP in blood donors and co-infection of HBV and TP, given the fact that both are sexually transmitted pathogens. Our data showed that the overall prevalence of TP infection in blood donors was 0.21% in Southern China (138 out of 64,871 screened donations), which was slightly lower when compared to the results from Jinan, China (0.33%) [11]. In a previous study from the US, the prevalence of donations categorized as consensus positive of syphilis was 28.4/100,000 [17]. A study from Colombia reported the prevalence of donations with positive anti-TP tests was 1.86% [18]. The prevalence of TP infection among blood donors in Yemen and Kyrgyzstan was 2.4% and 3.6%, respectively [19,20]. In African countries, Zambia exhibited the highest TP prevalence at 40.5% [21], followed by Equatorial Guinea at 21.51% [22] and Angola at 20% [23] while relatively lower prevalence was identified in four nations: Cameroon at 8.1% [24], Ghana at 7.5% [25], Tanzania at 4.7% [26], and Ethiopia at 1.5% [27]. Meta-analysis revealed the pooled prevalence of syphilis among blood donors in Thailand was 0.42% [28]. Collectively, these data indicate that although the prevalence of TP varies largely in blood donors from different countries/regions, TP screening serves as a reliable surrogate marker to ensure blood safety by preventing transfusion-related syphilis transmission.

Our current study demonstrated that the confirmed positive rate of TP infection in female blood donors was higher than that in male donors. In each age subgroup, the seroprevalence of anti-TP remained higher in female donors compared to male donors, which was consistent with the results of a previous study [11]. Furthermore, the prevalence increased significantly with age suggesting that older donors bear a higher risk in terms of blood safety. One of the reasons why women bear a higher risk of being infected with TP is that unprotected sexual behavior renders them more susceptible to infection. It has been reported that females were more likely to be infected with sexually transmitted diseases (STDs) partly because of the different physiology and anatomy of the genital organs between both sexes [29]. Studies have confirmed that the male-to-female transmission rate was higher than the female-to-male transmission rate in certain STDs, such as HIV [29,30]. Our findings further demonstrated that the seroprevalence of TP infection was higher in individuals with a lower educational level, which is consistent with the result from previous investigation [31]. This phenomenon indicated that a higher educational level contributes to the enhanced awareness of health knowledge leading to lower risk of infection [32]. Given ongoing social development and increased population mobility, public health authorities should prioritize the dissemination of STD knowledge among populations with lower educational levels.

TP remains viable for several years at −78 °C, and blood samples from syphilis patients may still retain infectivity for up to 4 days when stored at 4 °C [33]. Previous studies have demonstrated that TP screening is not an effective surrogate marker for assessing the risk of other TTIs, given that the majority of TTI-marker-positive blood donations were not positive for TP. In addition, the co-infection rate of HBV/TP among US blood donors was as low as 1.5 per 100,000 donations [34,35]. However, risk factors for syphilis infection in blood donors are also shared by other blood-borne pathogens such as HBV [36,37]. A Chinese blood center reported that approximately 0.8% of HBsAg-positive blood donations were also tested reactive for TP [38]. In the present study, among 64,871 screened blood donations, 0.21% were confirmed as TP-positive but NAT-negative. Of these TP-positive donations, 56.5% were anti-HBc-positive, and 5.1% were diagnosed with OBIs. Additionally, sequence analysis revealed that various mutations of the HBV genome may contribute to the extremely low HBV viral loads and/or detection failure of HBsAg, collectively leading to these un-identified OBIs by routine screening in blood establishments, even by the most sensitive individual nucleic acid testing (ID-NAT). Our data also demonstrated that without anti-TP screening, the residual risk of OBI remained at a high level of 1:49,141, posing a potential threat to blood safety. These findings suggest that TP screening in blood donors may serve as an additional safeguard measure to exclude donations with undetectable OBIs. The observed co-infection of TP and HBV among blood donors is not unexpected, as these sexually transmitted infections share common risk factors.

Sexual transmission has become an important mode of HBV transmission [39,40]. A study by the American Red Cross reported that four out of nine recipients (44.4%) contracted HBV infection via blood donations from the sexual partners of HBV-positive donors [41]. Existing research demonstrates that anti-HBc prevalence increases with age, reflecting a higher cumulative risk of HBV exposure via sexual contact among blood donors [42,43]. In addition, 15 observational studies conducted in Western and Pacific nations have demonstrated that sexual risk factors, such as contact with a hepatitis virus-infected partner, correlate with elevated HBV/HCV infection risk in blood donors [44]. In the present study, among 138 confirmed TP-positive blood donations, 56.5% were anti-HBc-positive, and 5.1% were identified as OBI/TP co-infections. Such a high prevalence of HBV–TP co-infection and elevated anti-HBc seropositivity indicate that sexual contact may be one of the origins for the occurrence of OBIs. Our previous investigation of donor couples with chronic HBV infection found that seven out of 10 infected couples shared HBV sequence homology over 99%, supporting the hypothesis that HBV transmission occurs between partners predominantly via sexual contact [10]. Notably, three of these seven cases were classified as OBIs. Collectively, these findings, in combination with results from previous studies, could suggest that sexual exposure to HBV-infected individuals constitutes one of the primary sources of OBIs.

Our study has limitations. No epidemiological or behavioral information was collected from the enrolled donors, comparative analyses (e.g., TP-positive versus TP-negative donors), appropriate statistical measures and/or evaluation of the diagnostic or predictive performance of TP screening were not performed in the present study. These limitations render our findings mainly descriptive in nature and future study should focus on direct comparisons between TP-positive and TP-negative in the prevalence of HBV infections, particularly OBIs.

## 5. Conclusions

In summary, of 138 (138/64,871, 0.21%) TP-positive but NAT-negative donors screened from 64,871 blood samples, 78 (78/138, 56.5%) were anti-HBc-positive, and seven (7/138, 5.1%) exhibited OBIs that were not detected by routine screening assays. Several mutations were observed in S and the BCP/PC gene of the HBV genome, which may contribute to the extremely low HBV viral loads and/or failure in HBsAg detection by routine blood screening, collectively leading to OBIs. These findings demonstrate that TP screening has the potential to serve as an additional safeguard to exclude donations co-infected with TP/OBI, as indicated by a relatively higher prevalence of undetectable OBIs in TP-positive blood donors in our current study.

## Figures and Tables

**Table 1:** The sensitivity and specificity of the tests used in this study.

Markers	Detection Technology	Sensitivity (LOD: IU/mL)	Specificity (%)
HBsAg	ELISA (Bio-Rad)	0.025	99.28–99.94
HBsAg	ELISA (LivZon)	1.33	99.7
HBsAg	HBsAg II Quant	0.05	99.9
Anti-TP	ELISA (LivZon)	1 NCU/mL	99.27
Anti-TP	ELISA (WanTai)	0.6 NCU/mL	99.5
HBV DNA	MPX2.0 ID HBV NAT	2.3	NA
HBV DNA	qPCR	5	NA
HBV DNA	Nested PCR	10 *	NA
HBV DNA	Procleix^®^ Ultrio Elite^®^ Assay	ID-NAT: 4.3 discriminatory HBV: 4.5	NA

NA: not available. * Combined with a larger 2.5–5 mL plasma extraction volume, the LOD is 2–4 IU/mL.

**Table 2:** Socio-demographic characteristics of 138 TP-confirmed positive donations stratified by sex.

	Over All (%)	Male (%)	Female (%)	*p*
Total screened donations	64,871	42,101 (64.9)	22,770 (35.1)	
Number of TP-confirmed positive donations	138 (0.21)	82 (0.19)	56 (0.25)	0.104
First-time donors	121 (87.68)	69 (50)	52 (37.68)	0.04
Repeated donors	17 (12.32)	13 (9.42)	4 (2.90)	0.232
Number of donations with OBI	7 (5.07)	6 (7.31)	1 (1.78)	0.241
Age distribution				
18–30	22 (15.94)	17 (12.32)	5 (3.62)	0.118
30–45	56 (40.58)	29 (21.01)	27 (19.57)	
45–60	60 (43.48)	36 (26.09)	24 (17.39)	
Education level				0.091
>High school	34 (24.6)	24 (17.3)	10 (7.2)	
≤High school	104 (75.4)	58 (42.0)	46 (33.3)	
Anti-HBc distribution				0.706
Anti-HBc+	78 (56.5)	45 (32.6)	33 (23.9)	
Anti-HBc−	60 (43.5)	37 (26.8)	23 (16.7)	
Anti-HBs distribution				0.150
Anti-HBs+	88 (63.77)	48 (34.78)	40 (28.99)	
Anti-HBs−	50 (36.23)	34 (24.64)	16 (11.59)	

Abbreviation: HBV, hepatitis B virus. The *p* value denotes comparison between males and females.

**Table 3:** Frequency of HBV serological markers and HBV DNA in different age groups of 138 confirmed TP-positive donations.

Age (Years)	Number	Anti-HBc (−)%	Anti-HBc (+)%	HBV DNA (%)
<30	21	19 (90.47)	2 (9.53)	0 (0)
30–40	41	21 (51.22)	20 (48.78)	1 (2.44)
41–50	49	16 (32.65)	33 (67.35)	6 (12.24)
>50	27	4 (14.81)	23 (85.19)	0 (0)
Total (%)	138	60 (43.47)	78 (56.52)	7 (5.07)

**Table 4:** Serologic and molecular characteristics of 7 donations co-infected with OBI and TP.

Donor	Sex	Age	Vaccine	Sero-Marks					HBsAg ELISA(BR/LZ)	Ultrio Elite	MPX	BCP/PC	S	qPCR	Genotype/Serotype	S Mutations	BCP/PC Mutations
				HBsAg (IU/mL)	Anti-HBs (IU/L)	Anti-HBc	HBeAg	Anti-HBe									
TB23	M	45	y	−	>1000	+	−	+	−/−	−	−	+	+	0	B/adw2	I110M, T131N, M133T, F134L	T1719G, A1726C, A1752T, A1846T, G1896A
TB54	M	50	N	−	201.9	+	−	+	−/−	+	−	+	−	0		/	T1719G, A1726C, G1896A
TB73	M	48	N	−	−	+	−	−	−/−	−	+	−	+	0	B/adw2	C64Y, Q101R, F134L, V168A	/
TB84	M	45	N	−	−	+	−	+	−/−	−	−	+	−	0		/	T1719G, A1752T, A1846T, G1896A, C1913A
TB97	M	46		−	−	+	−	+	−/−	−	−	−	+	5.6	C/adrq+	Q101R, T113K, K122R, M133T, G145R	
TB103	F	44	N	−	−	+	−	−	−/−	−	+	+	−	6.8		/	T1719G, A1752T, A1846T, G1896A, C1913A
TB140	M	41	N	−	−	+	−	−	−/−	−	−	+	+	0	B/adw2	I110M, Q129H, Y161F	T1719G, A1762T, G1764A, A1846T, G1896A

None of these donations had elevated levels of alanine aminotransferase. Abbreviations: BR, Bio-Rad (ELISA); LZ, LivZon (ELISA). MPX, MPX 2.0 ID NAT (HBV); +, positive; −, negative. 0 indicates undetectable viral load. Only the S-positive samples could be genotyped.

## Data Availability

The data sets used and/or analyzed during the current study are available from the corresponding author on reasonable request.

## Abbreviations

HBV = hepatitis B virus; HBsAg, hepatitis B surface antigen; anti-HBc, hepatitis B core antibody; anti-HBs, hepatitis B surface antibody; OBI = occult hepatitis B infection; CHB = chronic hepatitis B; NAT = nucleic acid testing; TP = Treponema Pallidum; TPPA = Treponema Pallidum Particle Agglutination Assay; TRUST = Toluidine Red Unheated Serum Test; ELISA(s) = enzyme-linked immuno-absorbent assay(s); ECLI = electrochemiluminescence immunoassay; STDs = sexually transmitted diseases; WHO = World Health Organization; ID = individual donation; MP = mini pool; PCR = polymerase chain reaction; LOD = limit of detection; BCP/PC = basic core promoter/pre-core; MHR = major hydrophilic region; STD = sexually transmitted disease; RR = residual risk.
